# Selenoprotein Transcript Level and Enzyme Activity as Biomarkers for Selenium Status and Selenium Requirements in the Turkey (*Meleagris gallopavo*)

**DOI:** 10.1371/journal.pone.0151665

**Published:** 2016-03-23

**Authors:** Rachel M. Taylor, Roger A. Sunde

**Affiliations:** Department of Nutritional Sciences, University of Wisconsin, Madison, Wisconsin, United States of America; Oklahoma State University, UNITED STATES

## Abstract

The current National Research Council (NRC) selenium (Se) requirement for the turkey is 0.2 μg Se/g diet. The sequencing of the turkey selenoproteome offers additional molecular biomarkers for assessment of Se status. To determine dietary Se requirements using selenoprotein transcript levels and enzyme activities, day-old male turkey poults were fed a Se-deficient diet supplemented with graded levels of Se (0, 0.025, 0.05, 0.1, 0.2, 0.3, 0.4, 0.5, 0.75, 1.0 μg Se/g diet) as selenite, and 12.5X the vitamin E requirement. Poults fed less than 0.05 μg Se/g diet had a significantly reduced rate of growth, indicating the Se requirement for growth in young male poults is 0.05 μg Se/g diet. Se deficiency decreased plasma GPX3 (glutathione peroxidase), liver GPX1, and liver GPX4 activities to 2, 3, and 7%, respectively, of Se-adequate levels. Increasing Se supplementation resulted in well-defined plateaus for all blood, liver and gizzard enzyme activities and mRNA levels, showing that these selenoprotein biomarkers could not be used as biomarkers for supernutritional-Se status. Using selenoenzyme activity, minimum Se requirements based on red blood cell GPX1, plasma GPX3, and pancreas and liver GPX1 activities were 0.29–0.33 μg Se/g diet. qPCR analyses using all 10 dietary Se treatments for all 24 selenoprotein transcripts (plus SEPHS1) in liver, gizzard, and pancreas found that only 4, 4, and 3 transcripts, respectively, were significantly down-regulated by Se deficiency and could be used as Se biomarkers. Only GPX3 and SELH mRNA were down regulated in all 3 tissues. For these transcripts, minimum Se requirements were 0.07–0.09 μg Se/g for liver, 0.06–0.15 μg Se/g for gizzard, and 0.13–0.18 μg Se/g for pancreas, all less than enzyme-based requirements. Panels based on multiple Se-regulated transcripts were effective in identifying Se deficiency. These results show that the NRC turkey dietary Se requirement should be raised to 0.3 μg Se/g diet.

## Introduction

The current National Research Council (NRC) Se requirement for the turkey is 0.2 μg Se/g diet for growing turkeys at all stages [1]. In 1967, Scott and colleagues [2] reported that turkey poults fed a practical diet containing 0.08 μg Se/g diet, without supplemental vitamin E, grew poorly and developed gizzard myopathy. Supplementation with 0.2 μg Se/g diet (total: 0.28 μg Se/g) as selenite prevented both poor growth and gizzard myopathy; only 0.18 μg Se/g diet total Se was required if the diet was supplemented with 11 IU vitamin E/kg diet. Subsequent studies found that plasma Se and plasma glutathione peroxidase (GPX) activities were 15 and 25%, respectively, of levels in poults fed diets containing 0.23 μg Se/g diet regardless of vitamin E supplementation [3], and that 0.13–0.17 μg Se/g diet or more was necessary to maximized plasma GPX activity [4].

These dietary Se requirements to prevent disease, maintain growth, and maximize GPX activity in the turkey stand out relative to other species. In rats, there is no dietary Se requirement for growth in today’s rapidly growing male rat pups, and 0.1 μg Se/g diet maximizes plasma and liver selenoenzyme activities [5]. In broiler chicks, 0.1 μg Se/g diet prevents poor growth and pancreatic atrophy [6] and 0.12 μg Se/g diet maximizes plasma GPX activity [7]. In lambs, 0.05 μg Se/g diet is sufficient for growth and 0.1 μg Se/g diet is required for maximum GPX activity [8]. In more recent studies with young pigs fed a basal diet without supplemental vitamin E and only 0.03 μg Se/g diet, there is no effect of supplemental Se on growth, 0.1 μg Se/g diet is sufficient to maximize plasma GPX3 activity, and 0.2 μg Se/g diet was required to maximize liver GPX1 activity [9]. Thus we began studying selenoenzyme expression in the turkey to better understand Se requirements, and found that at least 0.2 μg Se/g diet was required to maximize plasma and liver GPX activity [10].

Using today’s rapidly growing commercial poult and corn-soy diets, Fisher et al. [11] reported in 2008 that the Se requirement is 0.3 μg Se/g diet as selenate, based on achieving maximum tissue Se concentration and liver and plasma GPX activity. Using semi-purified diets with a basal Se content of 0.007 μg Se/g, Sunde and Hadley [12] showed that 0.05 μg Se/g was required for maximal growth and that 0.3 μg Se/g diet as selenite was required for both maximal GPX1 and GPX4 activities in liver and gizzard [12]. Clearly there is a need to update the NRC requirement for Se, as well as other avian nutrient requirements [13].

Transcriptomics offer the potential of discovering additional molecular biomarkers for assessment of Se status and requirements as well as better understanding the role of Se in disease [14,15]; molecular biomarkers are mRNA transcripts that respond to nutrient status of an animal or tissue. In mice and rats, we previously found that Se deficiency dramatically down-regulates the levels of GPX1, SEPW1 and SELH transcripts and that these biomarkers can be used to determine minimal Se requirements [16]. Interestingly, GPX4 mRNA as well as the majority of the 24 rodent selenoprotein transcripts are not regulated by Se status in rodents [5,17,18]. Furthermore, supernutritional-Se status (8X Se requirement) does not further up- or down-regulate any selenoprotein transcripts in rats, indicating that selenoprotein transcripts in rodents cannot be used as biomarkers of supernutritional-Se status [19]. In a previous study, we partially cloned turkey GPX1 and GPX4 mRNA and found that both were down-regulated in Se deficiency to 30% of Se-adequate levels [12], and we have now cloned and sequenced the 24 turkey selenoprotein transcripts [20], providing tools to study selenoprotein transcripts as potential biomarkers of Se status in the turkey.

The objectives of this study are: 1) to determine the Se requirement of a modern commercial strain of male turkey poults based on tissue selenoprotein enzyme activity; 2) to identify which selenoprotein transcripts are regulated by Se status and to use these as molecular biomarkers to assess Se status and Se requirements; 3) to evaluate selenoprotein mRNA for potential as biomarkers of supernutritional-Se status. In addition, the results allow development of panels of molecular biomarkers for assessment of Se status, and may generate hypotheses as to why different organs in different species are first-affected in Se deficiency.

## Methods

### Reagents

Molecular biology reagents were purchased from Promega (Madison, WI, USA), Invitrogen (Carlsbad, CA, USA), or Sigma (St. Louis, MO, USA). All other chemicals were of molecular biology or reagent grade.

### Animals and diets

Day-old male Nicholas white-derived turkey poults (kindly donated by Jennie-O Turkey Store, Barron, WI) were allocated randomly to treatment and housed in battery cages (5–6 per cage) with raised wire floors and 24-h lighting in the UW Poultry Research Laboratory, following the care and treatment protocol approved by the Institutional Animal Care and Use Committee at the University of Wisconsin (Protocol No. A01146). The temperature was set at 95°F for the first week and lowered 5°F in each subsequent week. Animals were fed from stainless steel troughs ad libitum, and allowed free access to deionized water in plastic waterers. The basal Se-deficient torula yeast-based diet (**Table 1**) was modified from diets we have used previously [12]. The diet was supplemented with 7.0% crystalline amino acids, including 0.93% L-methionine, to better match NRC recommendations for protein and amino acids. Vitamin E (as all-rac-α-tocopherol acetate) was supplemented at 150 mg/kg (12.5X NRC requirement), and levels of all other vitamins and minerals were supplemented to provide ~150% of the requirements [1]. The torula yeast was from the same lot as used in previous 30% torula yeast rodent studies where the analyzed Se content was 0.005 μg Se/g [5], and amino acids were primarily from the same lots of crystalline amino acids used for diets containing by analysis 0.003 μg Se/g diet [21]. The basal diet was supplemented with ten graded levels of Se (0, 0.025, 0.05, 0.1, 0.2, 0.3, 0.4, 0.5, 0.75. 1.0 μg Se/g diet) as Na_2_SeO_3_. Body weight was measured twice weekly. Extra birds were used in each treatment in case of mortality issues, although survivability in this experiment was very good. Over the course of the experiment, one bird was found dead and one had to be euthanized because of injury.

**Table 1:** Basal torula yeast-based poult diet^a^.

Ingredient	% of Diet
Torula yeast	30.000
Crystalline L-amino acids^b^	7.000
Sucrose	44.845
Lard	5.000
Mineral mix^c^	5.000
Vitamin mix^d^	0.900
Choline	0.210
Vitamin E^e^	0.015
Dicalcium phosphate	1.100
Calcium carbonate	0.750
Zn Mn supplement^f^	0.500
Solka-floc	4.680
**Total:**	100.000

^a^Provides 2899 kcal ME/kg diet

^b^L-amino acids (g/kg diet): Alanine, 2.75; Arginine, 7; Asparagine, 2.1; Aspartate, 2.75; Glutamate, 13.5; Glycine, 2.75; Histidine, 2.3; Isoleucine, 2.75; Leucine, 5.7; Lysine, 2.75; Methionine, 9.3; Phenylalanine, 2.75; Proline, 2.2; Serine, 2.75; Threonine, 2.75; Tryptophan, 0.95; Tyrosine, 1.65; Valine, 3.3

^c^Mineral mix (mg/kg diet): CaCO_3_, 3808.35; MgCO_3_, 175; MgSO_4_-7H_2_O; 112; NaCl, 483; KCl, 756; KH_2_PO_4_, 1484; (FeNH_4_)_3_-(citrate)_4_, 143.5; KI, 0.28; MnSO_4_-1H_2_O, 23.31; NaF, 7; Al(NH_4_)(SO_4_)_2_-12H_2_O, 1.12; CuSO_4_-5H_2_O, 6.3; Na_2_MoO_4_-2H_2_O, 0.07; NiCl_2_-6H_2_O, 0.07

^d^Vitamin mix (mg/kg diet): glucose monohydrate, 8752.5; thiamin HCl, 4.0; riboflavin, 2.5; pyridoxine HCl, 2; Ca-D-pantothenate, 20.0; niacin, 100.0; menadione, 1.0; folic acid, 2.0; biotin, 1.0; vitamin B-12 (0.1% trit), 10.0; retinyl palmitate (250,000 IU A/g), 100.0; cholecalciferol (400,000 IU D3/g), 5.0.

^e^DL-alpha-tocopherol acetate (Sigma T3376).

^f^Zn Mn supplement (mg/kg diet): (Zn(OH)_2_)_3_(ZnCO_3_)_2_, 110.0; MnSO_4_-1H_2_O, 90.0, sucrose, 4800.0.

### Tissue collection

The poults (5 per treatment) were killed at 28 days by terminal CO_2_ overexposure followed by exsanguination. Blood was collected in heparin-coated tubes, centrifuged (1500X g, 15 min, 4°C, Eppendorf 5415R, F-45-24-11 rotor, Brinkmann, Westbury, NY) to separate plasma from red cells (RBC), and RBC were reconstituted to original volume using saline phosphate buffer (76 nmol/L NaCl, 50 nmol/L sodium phosphate, pH 7.4). Liver, gizzard, and pancreas were collected and immediately frozen at -80°C until analysis. On tissue collection, one bird from the 0 μg Se/g diet group was found to be female and was omitted from statistical analysis.

### Enzyme activity analysis

Liver, gizzard and pancreas were homogenized in 9 vol of sucrose buffer (20 mmol/L tris-HCl, pH 7.4, 0.25 mmol/L sucrose, 1.1 mmol/L EDTA, 0.1% peroxide-free Triton-100) and centrifuged (10,000X g, 15 min). GPX1 activity in the RBC, liver, gizzard and pancreas, and GPX3 in the plasma were measured by the coupled assay procedure [22] using 120 μmol/L H_2_O_2_. GPX4 activity in the liver, gizzard and pancreas was measured by the coupled assay procedure [17] using 78 μmol/L phosphatidylcholine hydroperoxide. In liver, gizzard and pancreas, GPX1 specific activity was calculated by subtracting the activity due to GPX4, as described previously [12]. In both assays, 1 enzyme unit is defined as the amount of enzyme that will oxidize 1 μmol/min GSH under the specified conditions. Thioredoxin reductase (TXNRD) was measured using the gold-inhibition assay with DTNB (Sigma D8130) as the substrate, as described previously [23]. The protein concentration was determined by the method of Lowry et al. [24].

### RNA isolation and analysis

Total RNA from liver, gizzard and pancreas (~100 mg tissue, n = 5/group) was isolated using the guanidinium isothiocyanate method with TRIzol Reagent (Invitrogen, catalog no. 15596–026) following the manufacturer’s protocol. The RNA pellet was dissolved in 300 μl diethylpyrocarbonate-treated water and quantitated using a ND-1000 UV-Vis spectrophotometer (NanoDrop Technologies, Wilmington, DE). Relative mRNA abundance was determined by quantitative real-time PCR (qPCR). RNA (1 μg) was reverse transcribed to cDNA using the RETROscript kit (AM1710, Ambion) following the manufacturer’s protocol, and diluted 1/50 to make cDNA working stocks. Turkey gene-specific primers were based on the recently sequenced turkey selenoproteome [20] and designed to span an intron-exon splice junction and amplify a ~130 base segment [20]. The final 25 μl reactions contained 10 μl reverse transcribed cDNA working stock, 0.2 mmol/L turkey gene-specific forward and reverse primers, and 1X SybrGreen PCR Mastermix (Applied Biosystems no. 4309155). Reactions were followed in an ABI Prism 7000 (Applied Biosystems) with initial stages of 50°C for 2 min and 95°C for 10 min, followed by 40 cycles of 95°C for 15 sec and 60°C for 2 min. Dissociation curves were examined for each transcript to confirm the production of a single product. The amplification efficiency for each transcript was determined using the DART-PCR program [25]. The mRNA relative abundance was calculated according to Pfaffl [26], accounting for gene-specific efficiencies, normalized to the mean of β-actin (ACTB) and glyceraldehyde-3-phosphate dehydrogenase (GAPDH) expression, and expressed as a percentage of the plateau of Se-adequate levels. To compare transcript expression of different selenoproteins, relative abundance was normalized for basepair length of the amplified fragment.

### Statistical analysis

Data are presented as mean±SEM. For growth and enzyme analysis, n = 5 and for mRNA expression, n = 4–5 (in 0 μg Se/g diet group, n = 4). Data were analyzed by one-way ANOVA and variance equality was tested using Levene’s test for homogeneity of variances [27]. When the main effect of diet was significant, differences between means were assessed by Duncan’s multiple range analysis (*P*<0.05) with Kramer’s modification for unequal class sizes when necessary [28]. Regression equations for growth rate were calculated by the method of least squares for the relationship of Se supplementation and growth rate. For growth rate, differences between slopes of the regression equations were tested using analysis of covariance (ANCOVA) and Student-Newman-Keuls posttests (*P*<0.05). A “Se-response curve” was constructed using sigmoidal or hyperbolic regression analysis (Sigma Plot, Jandel Scientific) on all individual values at each dietary Se treatment, as described previously [5,29,30]. The “plateau breakpoint” for each Se-response curve, defined as the intersection of the line tangent to the point of steepest slope and the plateau, was calculated as described previously [5] to estimate the minimum dietary Se requirement necessary to obtain the plateau response.

### Biomarker panels

For Se-regulated selenoprotein transcripts in liver, gizzard, and pancreas, the relative selenoprotein mRNA expression levels for each individual poult, normalized and expressed relative to Se-adequate plateau as described above, were averaged to create a composite panel value for each poult [14,18]. These composite panel values were subjected to the same statistical analysis as the expression values for individual selenoprotein transcripts to evaluate the usefulness of transcript panels for assessment of Se status.

## Results

### Animal growth

**Fig 1:**
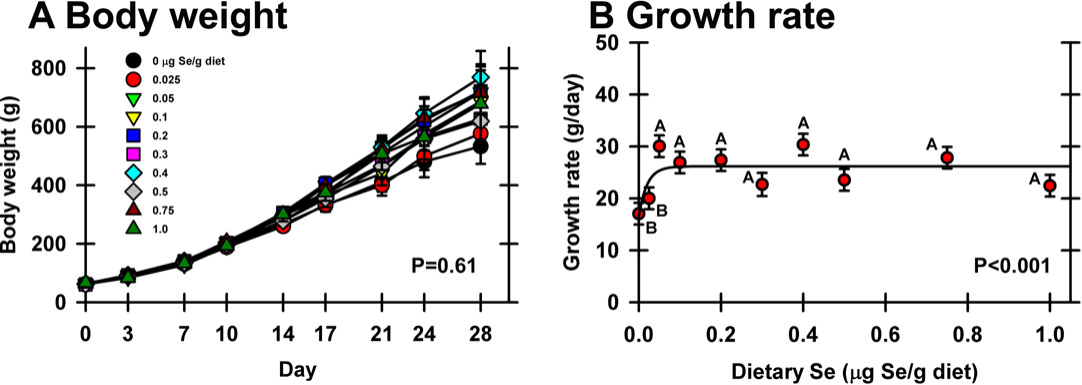
Effect of dietary Se on growth of young male poults. A. Body weights of day-old male poults supplemented with graded levels of dietary Se for 28 d (n = 5/treatment) and weighed biweekly. B. Daily weight gain of poults over days 7–28, expressed as g/day. Values are mean±SEM. Means without a common letter are significantly different (*P*<0.05).

Initial body weights of the day-old poults averaged 62.5±4.9 g. At day 28 there was no significant effect of Se supplementation on final body weight, although Se-deficient birds (0 μg Se/g) averaged 70% of the weight of poults fed 0.4 μg Se/g diet (541±77 g vs. 769±201 g, *P* = 0.61, **Fig 1A**). These poults, supplemented with 150 mg/kg vitamin E (12.5X NRC requirement), were generally healthy and showed no gross signs of gizzard myopathy or other gross tissue abnormalities. There was, however, a significant difference in growth rate; from day 7 to day 28, the 0 and 0.025 μg Se/g diet groups had a significantly lower rate of growth as compared to all other groups (17.0 and 19.9 g/d, respectively, vs. an average of 26.4 g/d for all other groups, **Fig 1B**). There was no effect of Se treatment on liver or gizzard weight as a percentage of body weight (data not shown). Based on growth rate, the minimum dietary Se requirement in this study is 0.05 μg Se/g diet (**Table 2**).

**Table 2:** Se requirement hierarchy in turkey poults.

Biomarker	Minimum requirement	Extent of regulation	
**Growth**	**μg Se/g diet**	**%**^a^	***P*-value**
Final body weight	<0.01	n/a	0.61
Growth rate (7–28 d)	0.05	70	<0.001
**Enzyme activity**	**μg Se/g diet**	**%**^a^	***P*-value**
Liver TXNRD activity	0.07	18	<0.001
Gizzard GPX1 activity	0.18	14	0.01
Liver GPX4 activity	0.23	6.7	<0.001
Gizzard GPX4 activity	0.24	16	<0.001
Pancreas GPX4 activity	0.25	27	0.01
RBC GPX1 activity	0.29	37	<0.001
Plasma GPX3 activity	0.29	2.3	<0.001
Pancreas GPX1 activity	0.30	1.2^b^	0.006
Liver GPX1 activity	0.33	3.2	<0.001
**Liver transcript levels**	**μg Se/g diet**	**%**^a^	***P*-value**
Liver GPX1 mRNA	0.07	35	0.04
Liver SELH mRNA	0.07	19	0.04
Liver GPX3 mRNA	0.08	35	0.02
Liver SELU mRNA	0.09	29	0.04
Liver DIO1 mRNA^c^	0.05	32	0.15
Liver SEPP1 mRNA^c^	0.05	36	0.18
Liver GPX4 mRNA^c^	0.08	29	0.13
**Gizzard transcript levels**	**μg Se/g diet**	**%**^a^	***P*-value**
Gizzard SELH mRNA	0.06	34	0.002
Gizzard GPX3 mRNA	0.07	41	0.002
Gizzard GPX1 mRNA	0.14	46	<0.001
Gizzard GPX4 mRNA	0.15	46	0.01
**Pancreas transcript levels**	**μg Se/g diet**	**%**^a^	***P*-value**
Pancreas SEPP1 mRNA	0.13	57	0.03
Pancreas SELH mRNA	0.15	43	0.02
Pancreas GPX3 mRNA	0.18	29	<0.001
**Panel transcript levels**	**μg Se/g diet**	**%**^a^	***P*-value**
Liver	0.08	17	1.4x10^-3^
Gizzard	0.13	16	7.6x10^-7^
Pancreas	0.15	18	1.3x10^-5^

^a^Extent of regulation: percentage of Se-deficient as compared to Se-adequate plateau

^b^Extent of regulation compared to 0.4 μg Se/g diet group

^c^Liver transcripts included in calculating liver transcript panel values

### Enzyme activity analyses

**Fig 2:**
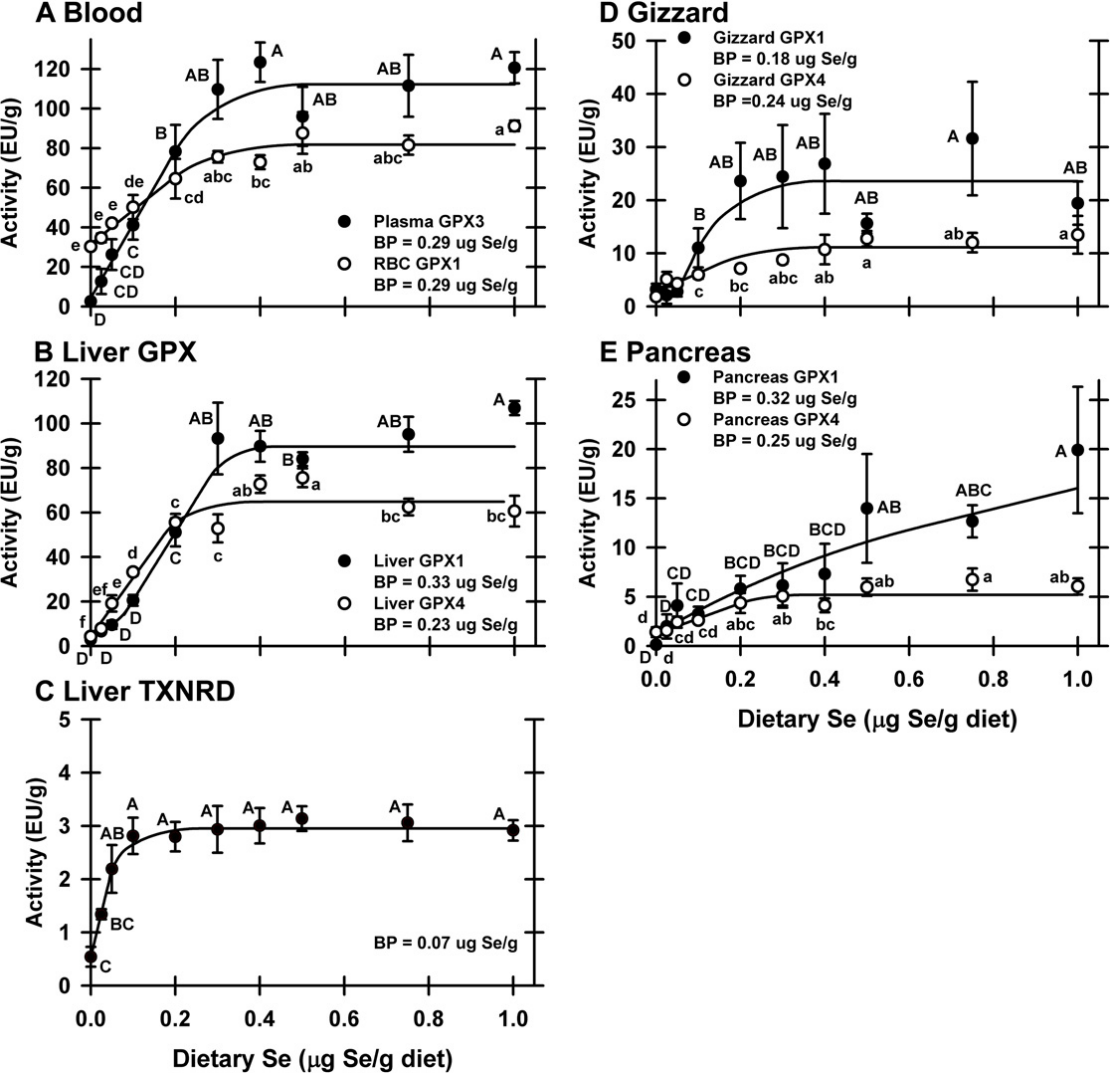
Effect of dietary Se on selenoenzyme activity. Activities for plasma GPX3 and RBC GPX1 (A), liver GPX1 and GPX4 (B), liver TXNRD (C), gizzard GPX1 and GPX4 (D), and pancreas GPX1 and GPX4 (E) in poults supplemented with graded levels of dietary Se for 28 d, expressed as enzyme unit (EU)/g protein. Values are mean±SEM. Means without a common letter are significantly different (*P*<0.05). Se-response curve breakpoints (BP) are indicated in each panel, calculated as described in the text.

Plasma GPX3 activity in 0 μg Se/g diet poults was 2.3% of the Se-adequate plateau, demonstrating that the birds were Se-deficient (**Fig 2A**). Graded dietary Se supplementation resulted in a hyperbolic response in plasma GPX3 activity with the plateau breakpoint at 0.29 μg Se/g diet. RBC GPX1 in Se-deficient poults was 37% of the plateau, and Se supplementation resulted in a hyperbolic Se-response curve, with the plateau breakpoint at 0.29 μg Se/g diet (**Fig 2B**). Both blood enzyme biomarkers rose linearly until reaching their respective breakpoints, with well-defined plateaus showing that supernutritional Se did not further increase enzyme activity.

Liver GPX1 activity in Se-deficient poults was 3.2% of Se-adequate levels. In liver, Se supplementation resulted in a sigmoidal response in GPX1 activity, with the breakpoint at 0.33 μg Se/g diet (**Fig 2B**). Liver GPX4 activity in Se-deficient poults fell to 6.7% of Se-adequate levels and rose hyperbolically with Se supplementation with the breakpoint at 0.18 μg Se/g diet (**Fig 2B**). TXNRD activity in Se-deficient poults was 18% of the Se-adequate plateau and rose hyperbolically with the breakpoint at 0.07 μg Se/g diet (**Fig 2C**).

In gizzard, GPX1 activity in Se-deficient poults fell to 14% of Se-adequate levels. This Se-response curve was sigmoidal as Se supplementation with 0.025 and 0.05 μg Se/g diet did not increase gizzard GPX1 activity, and then GPX1 increased linearly, with the plateau breakpoint at 0.18 μg Se/g diet (**Fig 2D**). Gizzard GPX4 activity was 16% of Se-adequate levels and Se supplementation resulted in a hyperbolic Se-response curve, with the breakpoint at 0.24 μg Se/g diet.

In pancreas, GPX4 activity in Se-deficient poults was 27% of Se-adequate levels, and Se supplementation resulted in a hyperbolic increase in GPX4 activity, with the plateau breakpoint at 0.25 μg Se/g diet (**Fig 2E**). In contrast to all other tissue selenoenzyme Se-response curves, Se supplementation did not result in a defined plateau for pancreas GPX1 activity. In Se-deficient poults, pancreas GPX1 activity was 1.2% of the level in poults supplemented with 0.4 μg Se/g diet. Supplementation with 0.025 and 0.05 μg Se/g diet resulted in the steepest increase in GPX1 activity; after 0.1 μg Se/g diet, GPX1 activity increased at 40% of the earlier rate such that GPX1 activity at 1.0 mg Se/g diet was double the level at 0.4 μg Se/g diet, with the plateau breakpoint at 0.32 μg Se/g diet.

Using selenoenzyme activity, the minimum dietary Se requirements based on RBC, pancreas and liver GPX1 activity, and plasma GPX3 activity were 0.29–0.33 μg Se/g diet in this study (**Table 2**). Se requirements based on liver, gizzard and pancreas GPX4 activity were 0.23–0.25 μg Se/g diet, underpinning these results. Thus the NRC dietary Se requirement should be raised to 0.3 μg Se/g diet.

### Selenoprotein mRNA abundance

**Fig 3:**
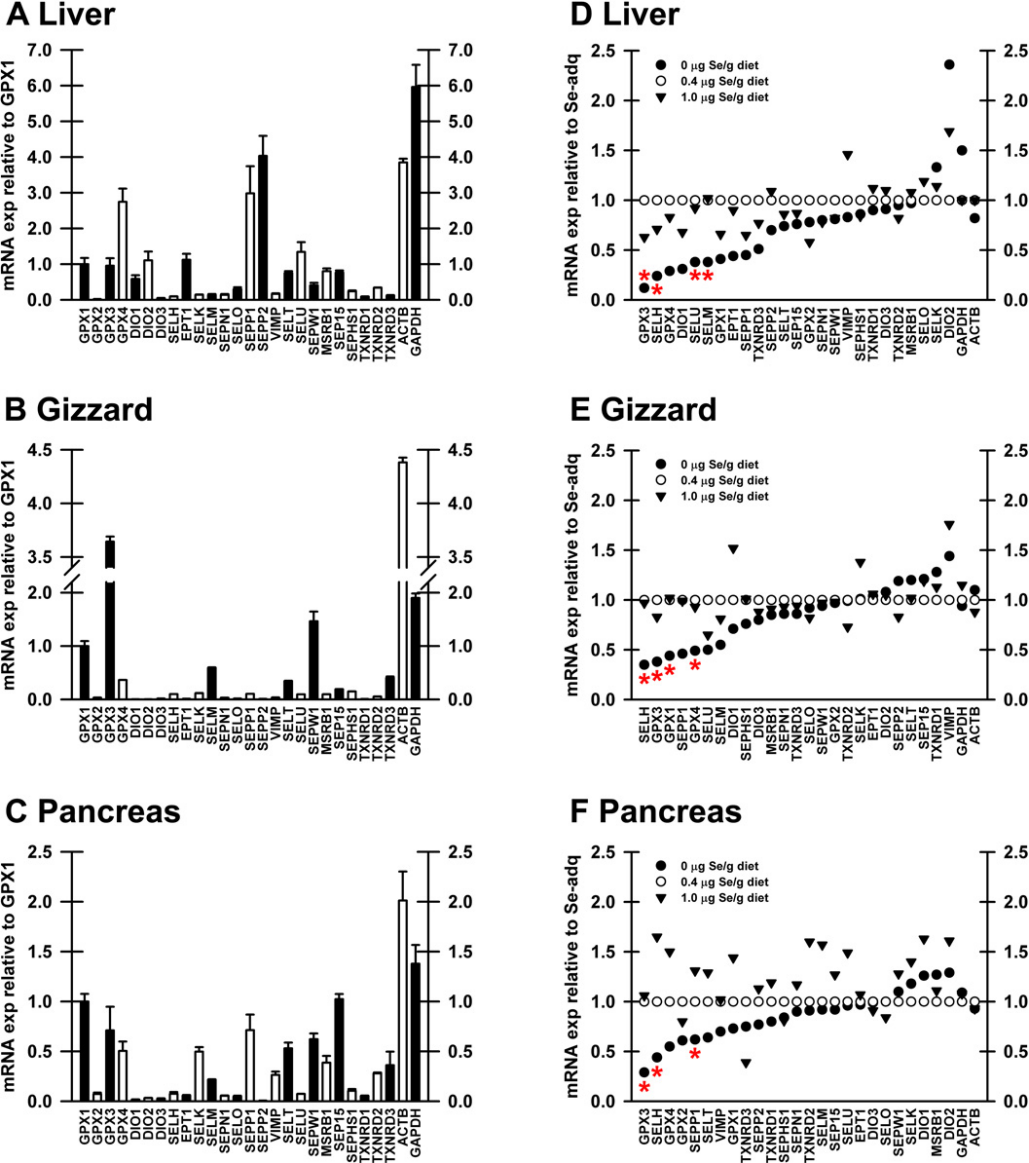
Relative expression of selenoprotein transcripts in liver (A,D), gizzard (B,E) and pancreas (C,F). A-C. Transcript expression for each selenoprotein, relative to GPX1 transcript expression, as described in text. Se-adequate tissues were from poults fed 0.4 μg Se/g diet. Bars show mean±SEM (n = 5). D-F. Pooled mRNA from poults supplemented with 0, 0.4 or 1.0 μg Se/g diet, expressed relative to transcript level in Se-adequate poults (0.4 μg Se/g diet). Red asterisks indicate significant effect of diet (*P*<0.05), as determined using all 10 Se treatments, as described in text.

To assess expression of the selenoprotein transcriptome, total RNA was isolated from liver, gizzard, and pancreas, reverse transcribed to cDNA, and analyzed by qPCR. We selected 0.4 μg Se/g diet as the Se-adequate level because enzyme activities in poults fed 0.4 μg Se/g diet were clearly on the plateau. Se-adequate samples were pooled to determine relative abundance of selenoprotein mRNA in Se-adequate tissues (**Fig 3A–3C**). For each tissue, GPX1 mRNA expression was set to 1 (arbitrary units). In liver, ACTB mRNA was 4X as abundant as GPX1, while GAPDH was approximately 6X as abundant as GPX1 (**Fig 3A**). SEPP1, SEPP2 and GPX4 mRNA in liver were expressed at levels comparable to ACTB, whereas GPX3 mRNA was expressed at the level of GPX1 mRNA. In Se-adequate gizzard, ACTB mRNA was 4.5X as abundant as GPX1 and GAPDH was 2X as abundant as GPX1 (**Fig 3B**). GPX3 mRNA in gizzard was more abundant than GAPDH mRNA and 4X as abundant as GPX1 mRNA, while GPX4 mRNA was half as abundant as GPX1 mRNA and SEPW1 mRNA was expressed at 1.5X the level of GPX1. In pancreas, ACTB mRNA was 2X as abundant as GPX1 mRNA and GAPDH mRNA was 1.5X as abundant as GPX1 mRNA (**Fig 3C**). Compared to liver and gizzard, the expression levels of selenoprotein mRNA in the pancreas were more uniform; SEP15, GPX3, SEPP1 and GPX4, and SELH mRNA were expressed at 1.0, 0.75, 0.75, 0.5 and 0.1X, respectively, of the level of GPX1 mRNA in pancreas.

### Se regulation of selenoprotein mRNA expression

To screen for Se regulation of transcript expression, Se-deficient, Se-adequate, and supernutritional-Se treatments (0.0, 0.4 and 1.0 μg Se/g diet, respectively), the five cDNA samples were pooled and assayed to determine transcript expression relative to the Se-adequate group (**Fig 3D–3F**). In this initial screen in liver, gizzard, and pancreas, 12 of 25 transcripts (24 selenoproteins plus SEPHS1) in each tissue were down-regulated in Se deficiency to ≤80% of the Se-adequate level (**Fig 3D–3F**, although not the same 12 genes in each tissue. Similarly, 2, 3, and 13 transcripts in liver, gizzard and pancreas, respectively, were upregulated with supernutritional Se to ≥120% of the Se-adequate level in the initial screen.

**Fig 4:**
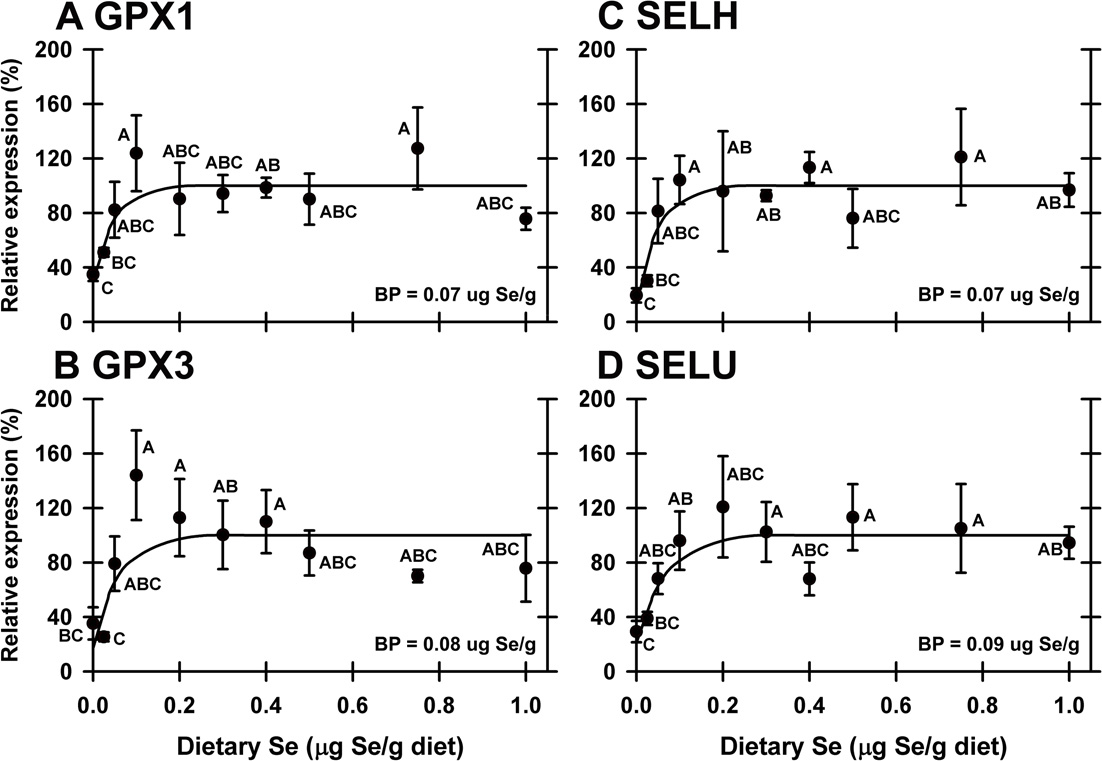
Effect of dietary Se on significantly-regulated selenoprotein transcripts in liver. Relative transcript levels are plotted for GPX1 (A), GPX3 (B), SELH (C) and SELU (D) in poults supplemented with graded levels of dietary Se for 28 d. Means without a common letter are significantly different (*P*<0.05). BP calculated as described in text. Overall level of significance given in Table 2.

**Fig 5:**
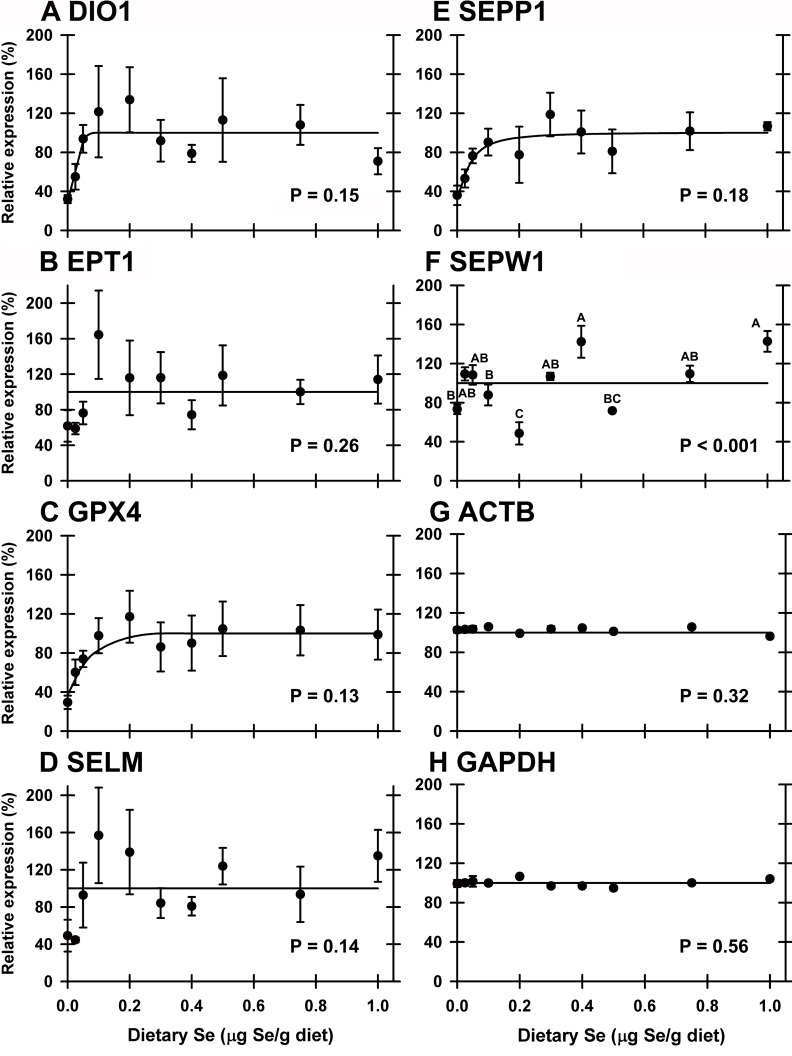
Effect of dietary Se on unregulated selenoprotein transcripts in liver. Relative transcript levels are plotted for DIO1 (A), EPT1 (B), GPX4 (C), SELM (D), SEPP1 (E), SEPW1 (F), ACTB (G) and GAPDH (H) in poults supplemented with graded levels of dietary Se for 28 d. Values are expressed as mean±SEM (n = 3–5/trt). Overall level of significance, as determined by ANOVA, indicated in each panel.

qPCR analyses of cDNA libraries from all 10 dietary Se treatments (n = 4–5 for each group) were performed on transcripts identified as potentially Se-regulated in the initial screen. In liver this analysis found that dietary Se significantly down-regulated expression of 4 of the 24 selenoprotein mRNA (GPX1, GPX3, SELH, SELU) (**Table 2**). Se-response curves were hyperbolic for these transcripts, and Se deficiency decreased transcript levels to 19–35% of plateau levels, with the breakpoints at 0.07–0.09 μg Se/g diet (**Fig 4A–4D**). SELH mRNA, which is poorly expressed in liver, was down-regulated to 19% of Se-adequate levels (**Fig 4C**). SELU mRNA, a selenoprotein not found in mammals [31], was expressed at levels comparable to GPX1 mRNA and was down-regulated to 29% in Se-deficient poults. In addition, GPX4, DIO1, and SEPP1 mRNA in liver were down-regulated to 29, 32, and 36%, respectively, of plateau levels, but these declines were not statistically significant (**Table 2; Fig 5A, 5C and 5E**). The remaining 18 selenoproteins were not significantly down-regulated by Se deficiency, including SELK and SEPW1, which have been shown to be highly down-regulated in liver by Se deficiency in the rodent [5,16]. No transcript in liver was significantly regulated by high Se when all 10 treatments were studied.

**Fig 6:**
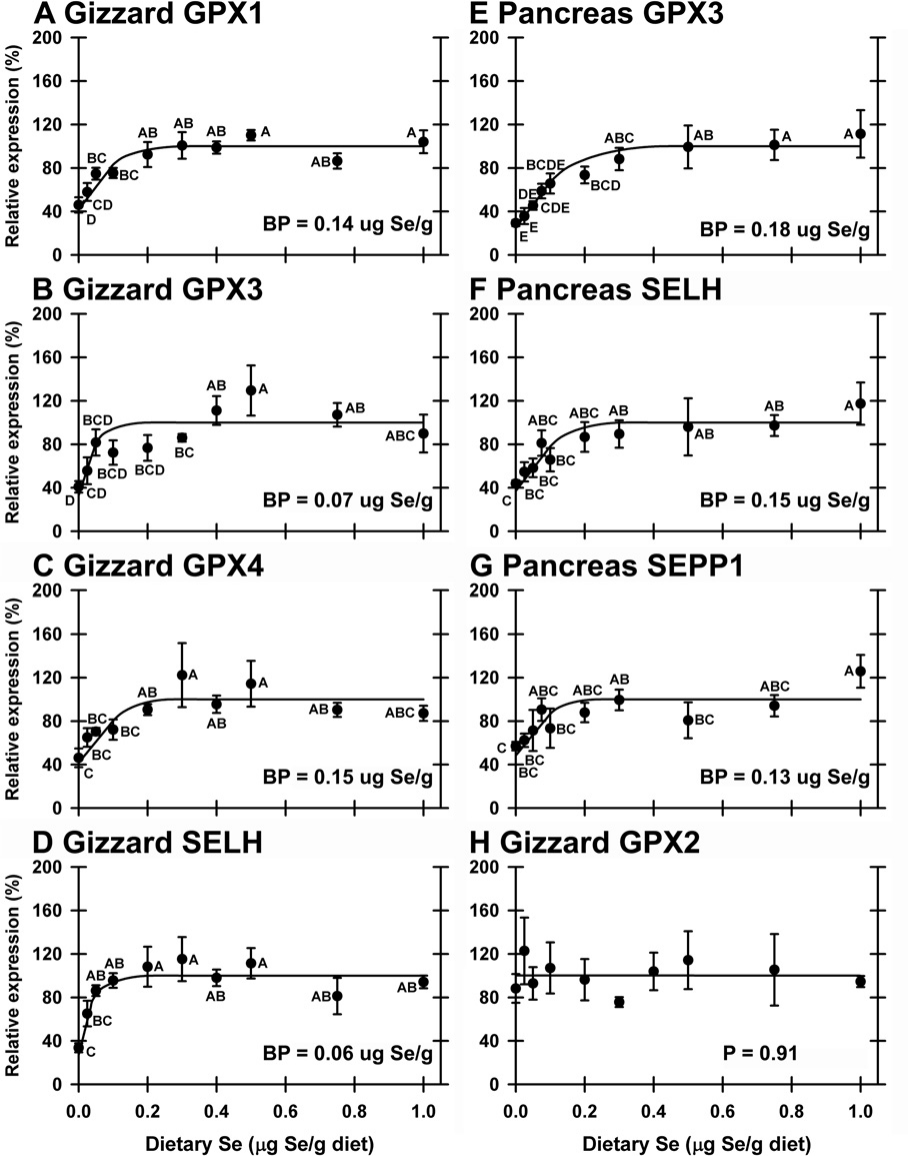
Effect of dietary Se on selenoprotein transcripts in gizzard (A-D, H) and pancreas (E-G). Relative transcript levels are plotted for gizzard GPX1 (A), gizzard GPX3 (B), gizzard GPX4 (C), gizzard SELH (D), pancreas GPX3 (E), pancreas SELH (F), pancreas SEPP1 (G) and gizzard GPX2 (H) in poults supplemented with graded levels of dietary Se for 28 d. Means without a common letter are significantly different (*P*<0.05). BP calculated as described in text. Gizzard GPX2 is displayed as example of an unregulated gene. Overall level of significance given in Table 2.

In gizzard, 4 of of the 24 selenoprotein mRNA (GPX1, GPX3, GPX4, SELH) remained significantly down-regulated in Se-deficient poults to 34–46% of Se-adequate levels when all 10 treatments were studied (**Table 2**). The resulting Se-response curves were hyperbolic, and GPX1 and GPX4 mRNA had breakpoints of 0.14–0.15 μg Se/g diet, whereas GPX3 and SELH had breakpoints of 0.06–0.07 μg Se/g diet (**Fig 6A–6D**). No transcripts in gizzard were significantly regulated by supernutritional-Se status.

In the pancreas, 3 of the 24 selenoprotein mRNA (GPX3, SELH and SEPP1) remained significantly down-regulated in Se-deficient poults (**Table 2**), with hyperbolic Se-response curves, decreasing to 29–57% of plateau levels, and with breakpoints of 0.13–0.18 μg Se/g diet (**Fig 6E–6G**). SEPP1 and GPX3 mRNA, the third and fourth most abundant transcripts in pancreas, and secreted selenoproteins in mammals [32,33], had breakpoints of 0.13 and 0.18 μg Se/g diet, respectively, whereas GPX1 mRNA was not significantly regulated in pancreas (*P* = 0.064), data not shown. When all 10 treatments were studied, no transcripts were significantly up or down-regulated by supernutritional Se (data not shown).

In these 3 tissues, the minimum Se requirements based on transcript biomarkers ranged from 0.06–0.18 μg Se/g diet. These minimal Se requirements are less than those based on selenoenzyme activity, just as observed for the rat [5]. There was no effect of Se treatment on GAPDH or ACTB transcript expression in any tissue (liver data shown in **Fig 5G and 5H**).

### Biomarker panels

**Fig 7:**
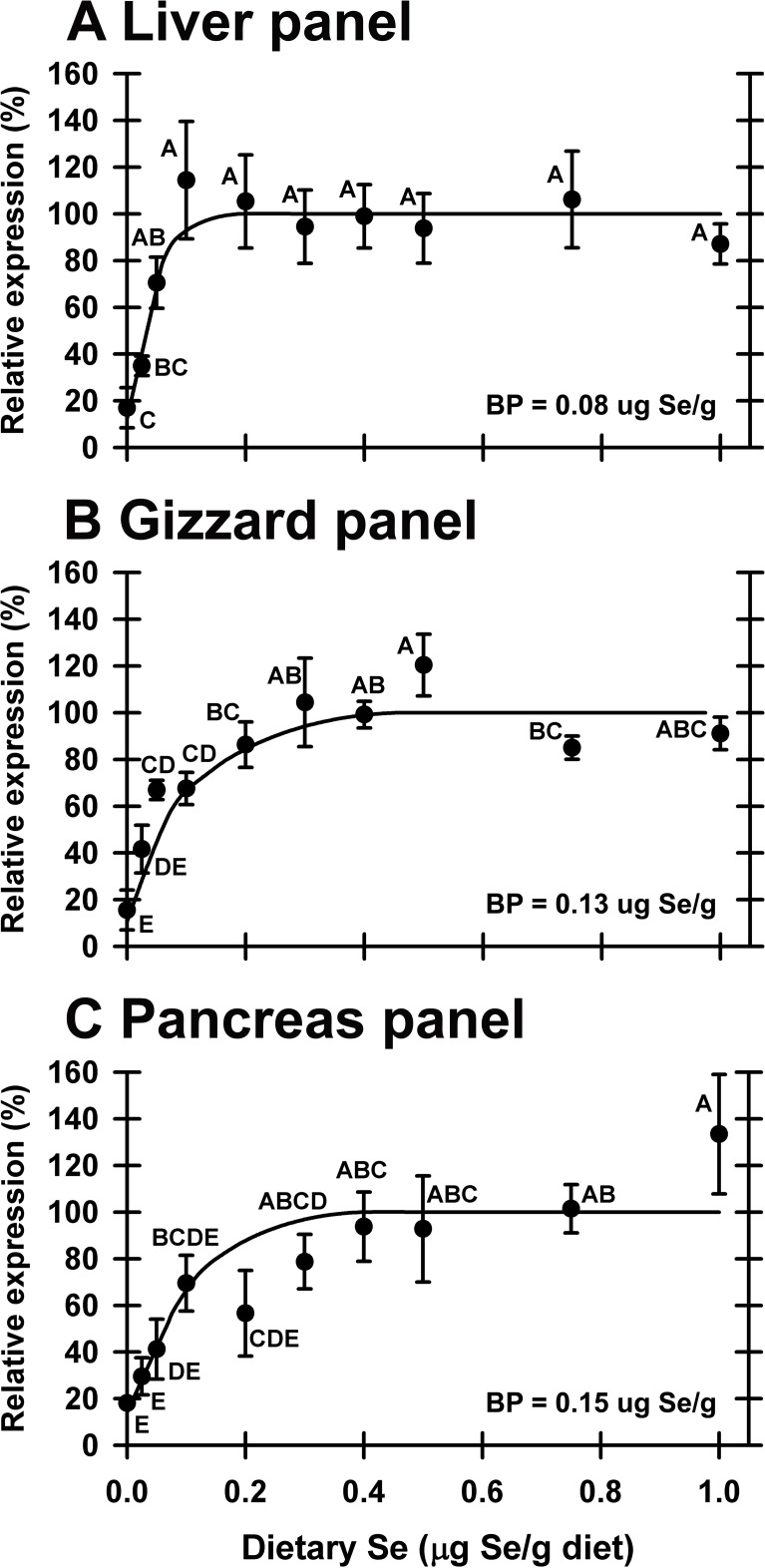
**Effect of dietary Se on selenoprotein transcript panels in liver (A), gizzard (B) and pancreas (C).** Within a tissue, the individual relative transcript levels for each significantly-regulated selenoprotein were averaged to determine panel values, which were then subjected to the Se-response curve analysis described in the text. Resulting mean±SEM are plotted, and means without a common letter are significantly different (*P*<0.05). Overall level of significance, as determined by ANOVA, is given in Table 2. Se-response curve breakpoints (BP) are indicated in each panel, calculated as described in text.

To better evaluate the usefulness of molecular biomarkers for assessing Se status, Se-response curves were generated using the composite panel biomarker values (**Fig 7**). When the 4 significantly-regulated transcripts in liver were used, the mean panel value for the Se-deficient group was 69% of Se-adequate levels. Inclusion of 3 additional transcript values (Table 2), which were decreased to the same extent as the significantly-regulated transcripts, increased the significance of the liver panel values substantially. For liver, gizzard and pancreas, the resulting Se-response curves were hyperbolic and more significant than the individual transcript response curves (*P* = 1.4x10^-3^, 7.6x10^-7^, 1.3x10^-5^ respectively, **Table 2**). Furthermore, the impact of Se deficiency was more pronounced in the panel Se-response curves, falling to 17, 16 and 18% of plateau levels, respectively, as compared to individual transcript response curves. This is likely because transcript levels in Se-deficient poults were consistently low for all genes, whereas individual poults fed Se-adequate diets with a low level for one transcript did not have uniformly low levels for the other transcripts.

## Discussion

In the present 28-day study, day-old male turkey poults were fed a Se-deficient diet supplemented with graded levels of Se up to 1.0 μg Se/g diet, and with 150 mg/kg vitamin E (12.5X NRC requirement). The poults were generally healthy, with no gross signs of gizzard myopathy, and there was no significant effect of Se-deficiency on final body weight or organ weight. Poults fed less than 0.05 μg Se/g diet, however, had a significantly reduced rate of growth, starting at day 7, indicating that the dietary Se requirement for growth in young male poults is 0.05 μg Se/g diet. Sunde and Hadley [12] also reported that the Se requirement for growth is 0.05 μg Se/g diet, using a similar semi-purified diet. Mueller et al. [34], using a corn-soy diet, reported that turkeys fed at least 0.05 μg Se/g diet grew normally. Our birds did not grow as rapidly as studies using corn-soy diets [11,34], but poults in the present experiment grew better than in our previous study [12] (day 28 average weight of Se-adequate poults 682.2 g vs. 536.7 g, respectively), most likely due to increased supplemental amino acids in the diet used in the present experiment.

In the plasma, RBC, and liver, GPX3 and GPX1 activities rose with increasing dietary Se to a well-defined plateau with breakpoints at 0.29, 0.29 and 0.33 μg Se/g diet, respectively, indicating that the minimum dietary Se requirement is 0.3 μg Se/g diet. This requirement is the same as recently reported for turkey poults fed vitamin E-adequate diets using blood GPX activity [11,12,34]. In the present study, only slightly less dietary Se (0.18–0.25 μg Se/g diet) was necessary for maximal levels of gizzard GPX1, and liver and gizzard GPX4 activities. The current NRC dietary Se requirement is 0.2 μg Se/g diet, based on growth and preventing gizzard myopathy in older studies [3,35,36]. In today’s commercial strain of rapidly growing male turkeys, these recent results indicate that the NRC turkey Se requirement should be raised to 0.3 μg Se/g diet.

In this study, Se deficiency had no gross effect on the gizzard, most likely because of the level of vitamin E and amino acid supplementation [37]. At the biochemical level, Se-adequate gizzard GPX1 and GPX4 activities were 25% and 15% of levels in liver, respectively, and declined in Se-deficiency to 15% of Se-adequate levels. Thus the apparent impact of Se deficiency in gizzard, based on GPX activity, is not as profound as in liver. Notably, the Se-response curve for gizzard GPX1 activity was quite sigmoidal, such that there was no increase in activity until 0.1 μg Se/g diet level; this suggests that limiting available Se is being incorporated into other, more important selenoproteins rather than into GPX1. Lack of Se for these other selenoproteins may be important in the development of gizzard myopathy.

Se requirements based on the Se-regulated transcriptome are almost uniformly lower than requirements based on enzyme activity. The present study found that only 4, 4, and 3 selenoprotein transcripts out of the 24 avian selenoproteins in liver, gizzard, and pancreas respectively, were significantly down-regulated in Se deficiency, whereas just as in rodents [5], the majority of the selenoprotein transcripts are not significantly down-regulated by Se deficiency in the turkey (**Fig 3**). Only GPX3 and SELH are down-regulated in all 3 tissues examined in the present study.

High expression of GPX4 activity and mRNA in the turkey may prevent the targeting of liver in Se deficiency in the avian. Just as in our previous study [12], turkey liver had very high GPX4 activity, and very low GPX1 activity, as compared to levels in rats [5]. In turkey liver, GPX4 mRNA only falls nonsignificantly (*P* = 0.13) to 29% of Se-adequate levels in Se deficiency, suggesting that GPX4 may be one of the selenoproteins that continues to protect turkey liver in Se deficiency. In rat liver, the organ targeted in Se deficiency in the rat, GPX1 mRNA is expressed at levels comparable to GAPDH, but falls in Se deficiency dramatically to 10% of Se-adequate levels; GPX4 mRNA is expressed in rat liver at 10% of GPX1 mRNA levels and little affected by Se deficiency [5]. In gizzard, in contrast, GPX4 mRNA levels are 33% of the level of GPX1 mRNA, and GPX4 activity in Se-adequate turkey gizzard is at the same level as in rat liver, suggesting that further declines of GPX4 levels may be associated with the targeting of gizzard in Se deficiency.

In turkey gizzard, GPX3 mRNA was expressed at almost twice the level of GAPDH mRNA, and in Se-deficiency, GPX3 mRNA falls significantly (*P* = 0.002) to 41% of Se-adequate levels with a breakpoint of 0.07 μg Se/g diet. The turkey GPX3 mRNA encodes a signal peptide for secretion, just as in mammals [33]. Thus the levels of GPX3 transcript in gizzard may enhance Se export, thus potentiating further reduction of gizzard Se level in Se deficiency.

The second highest expressed selenoprotein transcript in gizzard is SEPW1, a muscle selenoprotein in mammals, thought to be important in Se-dependent muscular dystrophy [38]. In turkey gizzard, there was no fall in SEPW1 transcript in Se deficiency, indicating that this mRNA is protected in Se deficiency. This suggests that this selenoprotein may be important in protecting gizzard in Se deficiency.

We have previously shown in rodents that panels of transcript biomarkers can be used to assess Se status and requirements [14,15]. When Se-response curves using liver panel values were constructed using seven regulated liver transcripts, the resulting plateau breakpoint (0.08 μg Se/g diet) was virtually the same as breakpoints determined using individual selenoprotein transcripts (**Table 2**). The mean value for Se-deficient poults (17%), however, was lower than means determined with individual transcripts, and significantly different from poults supplemented with 0.05 μg Se/g diet or higher, showing the power of a panel of biomarkers for identifying Se deficiency. This is likely because a Se-adequate poult with a low value for one transcript was not low for other transcripts, indicating that the poult was an outlier for this one mRNA and not truly Se-deficient. Biomarker panel analysis for gizzard had similar results, whereas the pancreas panel, comprised of 3 genes, was not as robust. These panels demonstrate that panels of several transcripts can be better biomarkers of nutrient status than single measures of mRNA abundance.

The present study was conducted at the same time as a study with day-old chicks fed virtually the same diets [39]. The growth rate of the Se-supplemented poults was half the rate of the chicks, with a minimum Se requirement for growth of 0.05 μg Se/g for poults vs. 0.025 μg Se/g for chicks. In poults, the minimum Se requirement based on plasma GPX3 and liver GPX1 activity was 0.3 μg Se/g, whereas in chicks the minimum Se requirement was 0.15 μg Se/g diet. In gizzard, GPX1 and GPX4 activities in the Se-adequate poult are 10 and 33% of activities in the Se-adequate chick, respectively. Relative to housekeeping genes, GPX3 mRNA in poult gizzard is not as high as in chick gizzard, but SEPP2 expression in poult liver is higher and SEPP1 in poult gizzard is lower than in the chick. These differences in GPX activities and expression of Se-transport transcripts may underlie the targeting of gizzard as the first affected organ in Se deficiency in the turkey but not the chicken.

In this study, and in our previous study [12], inorganic sodium selenite (Na_2_SeO_3_) was the form used for Se supplementation. Fischer et al. used sodium selenate (Na_2_SeO_4_) in their study with multiple Se levels [11]. All three studies concluded that 0.3 μg Se/g diet was the minimal dietary Se requirement. Additional forms of Se are commercially available as dietary supplements, including organic selenomethionine and related compounds. It would be of interest to evaluate Se biomarkers and Se requirements using graded levels of organic Se supplements to better understand their efficacy and metabolism.

This study was successful in developing molecular biomarkers to assess Se deficiency, but no selenoprotein mRNA was found to be significantly up- or down-regulated by supernutritional Se. It is likely, however, that expression of several non-selenoprotein transcripts will be affected by Se status [18,19]. One or more non-selenoprotein mRNA could serve as biomarkers of supernutritional Se, and additionally reveal clues to metabolic pathways potentially affected by Se status.

In conclusion, this study indicates that the turkey minimum Se requirement based on maximizing plasma GPX3, RBC GPX1, and liver GPX1 activities is 0.3 μg Se/g diet as selenite. Liver GPX4 activity and gizzard GPX1 and GPX4 activities were 0.18–0.24 μg Se/g diet, underpinning 0.3 μg Se/g diet as the Se requirement. The minimum Se requirement for growth of male turkey poults was 0.05 μg Se/g diet. This study, the first to use transcriptomics to explore the entire turkey selenoproteome, showed that a limited number of mRNA (4, 4, and 3 in liver, gizzard and pancreas, respectively) could be used as significant biomarkers of Se deficiency in today’s rapidly growing commercial poult. This study did not, however, detect any selenoprotein mRNA that could be used to determine supernutritional-Se status. This study demonstrates that Se requirements are 3X higher in turkeys as compared to rodents. Differential Se metabolism and regulation between tissues in the turkey may underlie the targeting of gizzard in Se deficiency. Given the variability in commercial turkeys, transcript biomarker panels have potential in precisely assessing Se status, and similar panels could be useful for other nutrients.
